# Finding Meaning in Medication Reconciliation Using Electronic Health Records: Qualitative Analysis in Safety Net Primary and Specialty Care

**DOI:** 10.2196/10167

**Published:** 2018-05-07

**Authors:** George Yaccoub Matta, Elaine C Khoong, Courtney R Lyles, Dean Schillinger, Neda Ratanawongsa

**Affiliations:** ^1^ Division of General Internal Medicine University of California, San Francisco San Francisco, CA United States; ^2^ UCSF Center for Vulnerable Populations Zuckerberg San Francisco General Hospital San Francisco, CA United States

**Keywords:** medication reconciliation, electronic health records, physician-patient relations, patient safety, communication

## Abstract

**Background:**

Safety net health systems face barriers to effective ambulatory medication reconciliation for vulnerable populations. Although some electronic health record (EHR) systems offer safety advantages, EHR use may affect the quality of patient-provider communication.

**Objective:**

This mixed-methods observational study aimed to develop a conceptual framework of how clinicians balance the demands and risks of EHR and communication tasks during medication reconciliation discussions in a safety net system.

**Methods:**

This study occurred 3 to 16 (median 9) months after new EHR implementation in five academic public hospital clinics. We video recorded visits between English-/Spanish-speaking patients and their primary/specialty care clinicians. We analyzed the proportion of medications addressed and coded time spent on nonverbal tasks during medication reconciliation as “multitasking EHR use,” “silent EHR use,” “non-EHR multitasking,” and “focused patient-clinician talk.” Finally, we analyzed communication patterns to develop a conceptual framework.

**Results:**

We examined 35 visits (17%, 6/35 Spanish) between 25 patients (mean age 57, SD 11 years; 44%, 11/25 women; 48%, 12/25 Hispanic; and 20%, 5/25 with limited health literacy) and 25 clinicians (48%, 12/25 primary care). Patients had listed a median of 7 (IQR 5-12) relevant medications, and clinicians addressed a median of 3 (interquartile range [IQR] 1-5) medications. The median duration of medication reconciliation was 2.1 (IQR 1.0-4.2) minutes, comprising a median of 10% (IQR 3%-17%) of visit time. Multitasking EHR use occurred in 47% (IQR 26%-70%) of the medication reconciliation time. Silent EHR use and non-EHR multitasking occurred a smaller proportion of medication reconciliation time, with a median of 0% for both. Focused clinician-patient talk occurred a median of 24% (IQR 0-39%) of medication reconciliation time. Five communication patterns with EHR medication reconciliation were observed: (1) typical EHR multitasking for medication reconciliation, (2) dynamic EHR use to negotiate medication discrepancies, (3) focused patient-clinician talk for medication counseling and addressing patient concerns, (4) responding to patient concerns while maintaining EHR use, and (5) using EHRs to engage patients during medication reconciliation. We developed a conceptual diagram representing the dilemma of the multitasking clinician during medication reconciliation.

**Conclusions:**

Safety net visits involve multitasking EHR use during almost half of medication reconciliation time. The multitasking clinician balances the cognitive and emotional demands posed by incoming information from multiple sources, attempts to synthesize and act on this information through EHR and communication tasks, and adopts strategies of silent EHR use and focused patient-clinician talk that may help mitigate the risks of multitasking. Future studies should explore diverse patient perspectives about clinician EHR multitasking, clinical outcomes related to EHR multitasking, and human factors and systems engineering interventions to improve the safety of EHR use during the complex process of medication reconciliation.

## Introduction

Clinicians in US safety net clinics—federally funded clinics serving socioeconomically disadvantaged populations [1]—face unique barriers to conducting effective ambulatory visit medication reconciliation. During medication reconciliation, as defined by the US Joint Commission National Patient Safety Goals, a clinician or care team member “compares the medications a patient should be using (and is actually using) to the new medications that are ordered for the patient and resolves any discrepancies” [2]. Although a requirement for safe transition of care, medication reconciliation is also an important patient-centered process for revealing patients’ knowledge, concerns, and behaviors around their medications that should inform treatment decision making and can affect adherence [3]. Limited evidence exists for the most effective interventions to integrate medication reconciliation into the workflow of ambulatory care [4]. Meanwhile, safety net patients with limited health literacy and limited English proficiency experience communication barriers that increase their risk of incorrectly reconciled medications and medication error [5-10].

Safety net systems could receive incentives to facilitate electronic health record (EHR) implementation costs by meeting metrics for medication reconciliation for EHR “meaningful use,” defined by the US Centers for Medicare and Medicaid Services (CMS) as “the process of identifying the most accurate list of all medications that the patient is taking, including name, dosage, frequency, and route, by comparing the medical record to an external list of medications obtained from a patient, hospital, or other provider” [11]. Although EHR use may improve patient-clinician communication if used to engage patients [12], EHR use may worsen communication by reducing eye contact, increasing silence and clinician multitasking, and shifting talk away from patient-centered topics [13-15]. Thus, EHR use may enhance or decrease patient-centered interviewing important to effective medication reconciliation.

Clinician multitasking—performing two or more tasks simultaneously [16]—may also affect medication reconciliation. Common examples of clinician EHR multitasking include eliciting a history while entering data (voluntary multitasking) or listening to a patient’s question while ordering a prescription (externally prompted multitasking) [16-17]. Multitasking may increase risk of errors, either in communication with patients, such as missing cues, or in completing EHR tasks, such as documentation or computerized order entry [16,18-20]. Technology-induced errors may arise if EHRs increase the clinician’s cognitive burden because of inadequate EHR design and development, problematic implementation and customization, or negative impacts on sociotechnical work processes [18-19]. If clinicians cope with this cognitive burden by using EHRs in silence, patients may be less satisfied [13,21]. However, delaying EHR use until later may not only risk errors because of potential memory lapses, but also increase clinicians’ EHR workload, stress, and burnout [22,23].

To our knowledge, no study has examined ambulatory safety net communication during medication reconciliation using newer EHRs certified for meaningful use. In a prior study, we found that safety net clinicians spent 30.5% of visits multitasking on EHRs, silently used EHRs 4.6% of visit time, and used 33.1% of visit time for focused patient-clinician talk [17].

We conducted a mixed-methods study using observations of real-world safety net ambulatory encounters to develop a conceptual framework of how clinicians balance the demands and risks of EHR and communication tasks during medication reconciliation discussions.

## Methods

### Study Design and Participants

We conducted this observational study in five primary and specialty care safety net clinics that had recently transitioned (range 3-16 months, median 9) from a “basic” EHR to a CMS-certified “fully functional” EHR [24]. In this ambulatory EHR, medication information was potentially documented in multiple areas:

1. “Current medications list” in the patient EHR chart and “current medications” section of visit notes. The active medication list in a patient’s electronic record is automatically imported into a visit note on the day of a visit for clinicians to update. Clinicians can check a box labeled “verified medications,” placing a phrase in the note “medication list reviewed and verified with the patient” and meeting the “meaningful use” CMS metric. At the time of this study, refill data from pharmacy claims was not available.
2. History of present illness. Clinicians narratively document the patient-reported medication concerns or behaviors.
3. Assessments and plan. Clinicians may narratively document references to patient concerns or behaviors influencing their decision making.
4. Treatment and orders. Rather than resolving discrepancies in the current medication list, some clinicians may adjust medications in the treatment section or type instructions for patients.

Eligible patients included English- or Spanish-speaking adults (age >18 years) with at least one of three chronic medical conditions (diabetes, congestive heart failure, or rheumatoid arthritis) who received primary care in the adult internal medicine or family medicine clinic *and* subspecialty care at a diabetes, cardiology, or rheumatology clinic [15,17,25]. Eligible clinicians included physicians, nurse practitioners, fellows, and residents. All participants provided written informed consent, and the Institutional Review Board of the University of California, San Francisco, approved the study.

### Data Collection

For participating dyads, we collected the following data from one clinician-patient visit [15,17,25]:

1. Structured previsit and postvisit interviews with patients to collect sociodemographic and medical data. Postvisit patient interviews occurred in person or via telephone. Native Spanish speakers translated and back-translated Spanish interview items into English.
2. Online questionnaires with providers to collect sociodemographic data.
3. Visit note written by clinician in the EHR.
4. Video recording of patient and provider visit.

### Data Measurements

#### Sociodemographic Information

We used previously validated self-report screening questions to determine patients’ English proficiency and health literacy [26-28]. We categorized Spanish-speaking patients who reported English proficiency less than “very well” as having limited English proficiency [26]. We categorized patients who were “somewhat,” “a little bit,” or “not at all” “confident filling out medical forms by yourself” as having limited health literacy [27,28].

#### Electronic Health Record Visit Documentation

We reviewed the EHR note corresponding to the videotaped interactions for the current medication list, any narrative text in any note section referring to medications, and the medications listed in the treatment section.

#### Number of Relevant Medications and Proportion Addressed

From the EHR visit note, we abstracted the number of medications on the EHR list or in the text of the visit note. Primary care providers are expected by national standards and local hospital policy to reconcile all medications, including any over-the-counter or nutritional supplements [3,11]. The local hospital policy specifies that specialty care medication reconciliation is required for “all medications related to their specialty, including those that may have drug or disease interactions” [29]. Thus, in addition to the total number of medications, we also created a category of “relevant medications.” Classified by a physician investigator (NR), we included the following medications (listed in the note and discussed during the visit) as relevant:

1. Primary care: all medications, including any over-the-counter or nutritional supplements.
2. Cardiology: all antihypertensive, diuretic, antiplatelet, lipid-lowering, antiarrhythmic, or pulmonary hypertension medications.
3. Rheumatology: all immunosuppressant or analgesic medications or medications to mitigate those regimens’ side effects (eg, folic acid with methotrexate or bisphosphonate with prednisone).
4. Diabetes: all oral or injectable hypoglycemic medications, antihypertensive medications, lipid-lowering medications, and aspirin. We excluded glucose monitoring supplies.

We considered a medication explicitly addressed if the patient or clinician specifically discussed its current or past use. We then summed the number of medications from the EHR medication list that were explicitly addressed during the visit, compared with both the number of relevant medications and the number of total medications.

#### Meaningful Use Indicator

We abstracted this if notes contained the phrase “medication list reviewed and reconciled with the patient.”

#### Medication Reconciliation Duration

We classified visit time as related to medication reconciliation during segments when clinicians or patients demonstrated behaviors to compare patients’ current medication-taking behaviors with the clinicians’ available medication lists or to elicit and respond to patients’ medication-related concerns and beliefs. We did not include discussions of newly prescribed or newly recommended medications. This duration included both verbal statements and nonverbal behaviors. Patient verbal statements included those elicited by clinicians’ questions or volunteered independently. Clinician verbal statements included those elicited by patients’ questions or volunteered independently. Nonverbal behaviors included clinicians’ visual inspections of patient medication bottles or paper medication lists as well as data entry or review using the EHR. We then calculated total visit minutes and proportion of visit time spent on medication reconciliation.

### Analysis

#### Electronic Health Record Use and Non-Electronic Health Record Behaviors During Medication Reconciliation

We categorized each segment of medication reconciliation time as mutually exclusive categories to describe whether clinicians conducted EHR-related or non-EHR-related tasks [17]: multitasking EHR use (clinician or patient speaking while clinician EHR use), silent EHR use (≥3 seconds silence), non-EHR multitasking (eg, paper chart, glucometer use), silent non-EHR use (≥3 seconds silence), and focused clinician-patient talk (no multitasking on EHR or non-EHR use).

#### Analysis of Communication and Electronic Health Record Use Patterns

We then conducted qualitative analysis to uncover patterns of communication and EHR use during medication reconciliation. We used a grounded theory approach, by which a theory to explain a phenomenon is derived from the data itself [30]. Two investigators (GYM and NR) independently analyzed a subset of videos, generating codes and negotiating discrepancies to create a preliminary coding template. One investigator (GYM) coded each remaining video with this template. Application of the template for coding and modifications to the coding template were arrived at by consensus. We conducted qualitative analysis using ATLAS.ti version 7.5.15 (Scientific Software Development GmbH, Berlin, Germany). We chose representative quotes highlighting these patterns. From these codes and patterns (Multimedia Appendix 1), we developed a conceptual framework of how clinicians balance the demands and risks of EHR and communication tasks during medication reconciliation discussions.

## Results

### Visits and Participants

We recorded 35 visits (17 primary and 18 specialty care) between 25 patients and 25 clinicians. Table 1 shows patient, clinician, and relationship characteristics. Patients were mean 57 (SD 11) years in age, 44% (11/25) were women, 48% (12/25) were Hispanic/Latino, and 20% (5/25) had limited health literacy. The majority reported that their health was “poor” (40%, 10/25) or “fair” (20%, 5/25). Among clinicians, most were women (72%, 18/25) and 48% (12/25) were primary care physicians (PCPs). Among the 35 visits, 51% (18/35) were in primary care, and 40% (14/35) reported receiving care from the clinicians for more than 5 years. The median visit length was 20.6 (interquartile range [IQR] 16.7-32.2) minutes, and 17% (6/35) were in Spanish.

### Task Performance and Medications Addressed During Medication Reconciliation

Table 2 describes the summary characteristics of medication reconciliation during each visit. The median duration of medication reconciliation was 2.1 minutes (interquartile range [IQR] 1.0-4.2 minutes), and medication reconciliation comprised a median of 10% (IQR 3%-17%) of visit time. EHR multitasking comprised a median of 47% (IQR 26%-70%) of medication reconciliation time. Silent EHR use and non-EHR multitasking occurred a smaller proportion of medication reconciliation time, with a median of 0% for both (IQR 0%-6% and IQR 0%-13%, respectively). Silent non-EHR tasks were not performed during medication reconciliation. Focused clinician-patient talk occurred a median of 24% (IQR 0%-39%) of medication reconciliation time. The median for total medications was 13 (IQR 9-17), with a median of 7 (IQR 5-12) relevant medications. The median number of addressed medications was 2 (IQR 1-5).

**Figure 1:**
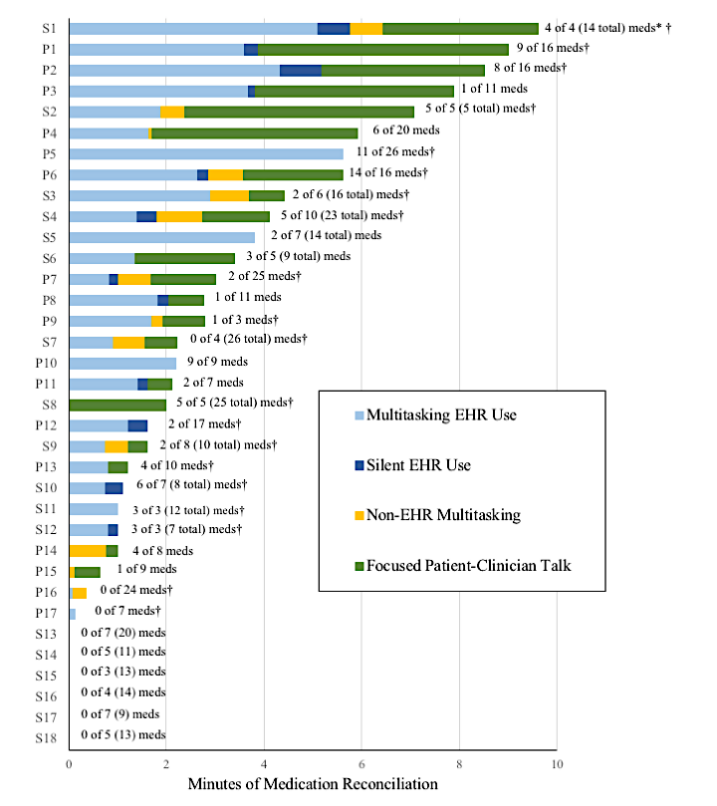
Multitasking, silent electronic health record (EHR) use, and number of medications explicitly addressed during safety net medication reconciliation (N=35). *Primary care encounters are labeled with a “P” and specialty care encounters with an “S.” The number following each line indicates the number of “addressed” medications out of the total number of “relevant” medications. Medications were categorized as “addressed” if the patient or clinician specifically discussed its current use. For primary care encounters, all medications listed in the patient’s note or discussed during the visit encounter were categorized as “relevant.” For specialty care encounters, medications related to the clinician’s specialty or with drug or disease interactions were categorized as “relevant”; the total number of all medications is listed in parentheses for these specialty encounters. † means clinicians clicked on a box labeled “verified medications” to indicate that medication reconciliation was performed.

Figure 1 depicts duration of medication reconciliation and the proportion of activities occurring during medication reconciliation for each visit, with primary care visits labeled as “P” and specialty care visits were labeled as “S.” The end of each bar is labeled with the number of medications explicitly addressed out of all relevant medications, with an asterisk indicating if the meaningful use medication reconciliation box was checked in the visit note. For specialty care encounters, the total of all medications is listed in parentheses. Among 35 visits, 29 (83%) involved a medication reconciliation discussion, almost always interspersed with other content and tasks throughout the visit, rather than in a single, uninterrupted segment. Clinicians multitasked on EHRs during medication reconciliation in 28 (80%) visits, with EHR multitasking occurring during the entirety of medication reconciliation in 5 (14%) of the visits. Silent EHR use occurred in 12 (34%) visits. The meaningful use medication reconciliation box was checked in 19 (54%) visit notes.

### Communication and Electronic Health Record Use Patterns During Medication Reconciliation

Five sets of communication patterns with EHR medication reconciliation were observed. Within a given visit, multiple patterns could have been observed.

#### Pattern 1: Typical Electronic Health Record Multitasking for Medication Reconciliation

In the most common pattern, clinicians reviewed and added information in the EHR current medication lists while talking with patients about their medications, as demonstrated in visit S11 (female rheumatologist, female patient, Spanish-concordant; 100% EHR multitasking; 1 minute):

[Clinician sits facing the EHR current medication list, with the patient adjacent to the monitor, facing the clinician.]

Clinician: “So you’re taking Enbrel every week?”

Patient: “Yes.”

Clinician: [clicks box checked, scrolls] “Okay, and you’re still taking hydroxychloroquine—”

Patient: “Yes.”

Clinician: “—once a day.”

Patient: “It’s once now?”

Clinician: [shifts gaze to patient for <1 second, then back to EHR] “You’re taking it twice?”

Patient: “Yeah, it was like that.”

Clinician: [clicks on medication in list, clicks to change frequency] “Okay, okay.”

**Table 1:** Patient, clinician, and visit characteristics in a study of electronic health record use in safety net primary and specialty care medication reconciliation.

Characteristics			Value
**Patients (n=25)**			
	Age (years), mean (SD)		56.8 (11.0)
	Gender (female), n (%)		11 (44)
	**Race/ethnicity, n (%)**		
		Hispanic	12 (48)
		Asian	6 (24)
		Caucasian	4 (16)
		African-American	2 (8)
		Multiethnic	1 (4)
	**Language, n (%)**		
		Primary language Spanish	10 (40)
		Limited English proficiency	6 (24)
	**Education, n (%)**		
		≤8th grade education	2 (8)
		Some high school or high school graduate/General Education Diploma	7 (28)
		Some college or college graduate	16 (64)
	Limited health literacy, n (%)		5 (20)
	Income (≤US $20,000/year), n (%)		23 (92)
	“Poor” or “fair” quality of life, n (%)		18 (60)
**Clinicians (n=25)**			
	Age (years), mean (SD)		44.9 (11.9)
	Gender (female), n (%)		14 (67)
	**Clinic, n (%)**		
		Primary care clinic	14 (56)
		Diabetes clinic	5 (20)
		Cardiology clinic	3 (12)
		Rheumatology clinic	3 (12)
	**Role, n (%)**		
		Physician	21 (84)
		Nurse practitioner or physician assistant	4 (16)
	Years since professional degree, mean (SD)		15.7 (11.3)
**Visits (n=35)**			
	**Relationship length years at baseline, n (%)**		
		<1 year	2 (6)
		1-5 years	19 (54)
		>5 years	14 (40)
	**Language during visit, n (%)**		
		English	29 (83)
		Spanish	5 (14)
		Spanish interpreter	1 (3)
	Visit length (minutes), median (interquartile range)		20.6 (16.7-32.2)

**Table 2:** Characteristics of medication reconciliation during safety net primary and specialty care visits (n=35).

Medication reconciliation characteristics		Value	
Medication reconciliation duration (minutes), median (IQR^a^)		2.1 (1.0-4.4)	
**% of medication reconciliation time spent performing activities, median (IQR)**			
	Multitasking EHR^b^ use		47 (26-70)
	Silent EHR use		0 (0-6)
	Non-EHR multitasking		0 (0-13)
	Focused patient-clinician talk		24 (0-39)
Number of total medications^c^, median (IQR)		13 (9-17)	
Number of relevant medications^c^, median (IQR)		7 (5-12)	
Number of relevant medications addressed^d^, median (IQR)		2 (1-5)	

^a^IQR: interquartile range.

^b^EHR: electronic health record.

^c^The total medications included all listed in the patient’s note or discussed during the visit encounter. All medications were categorized as relevant for primary care encounters. For specialty care encounters, relevant medications were related to the clinician’s specialty and those with drug or disease interactions.

^d^Medications were categorized as “addressed” if the patient or clinician specifically discussed its current use.

Occasionally this also included interspersed EHR multitasking with non-EHR multitasking, such as looking at pill bottles or paper medication lists to update the EHR current medication list. In encounter P11 (male PCP, male patient, English-concordant; 68% EHR multitasking, 9% silent EHR, 24% focused clinician-patient talk; 2.1 minutes) a clinician used the EHR and pill bottles to check a recently uninsured patient’s medications:

[Clinician sits facing EHR screen. Patient sits adjacent to the screen, facing clinician, and takes pill bottles out of bag.]

Clinician: [holds bottle in hand, looks at label for 1 second] “Are you taking these—” [puts down bottle, looks at EHR current medication list] “—every day or only once in a while?”

Patient: [takes out other pill bottles] “Once in a while.”

Clinician: [begins typing into medication list] “Okay.”

Patient: [shakes bottle] “Uh, I’m down to one here.”

Clinician: [glances at pill bottle for <1 second, then to EHR] “Okay, would you say all of these you’re taking just once in a while?” [looks at bottle for <1 second then to EHR]

Patient: “Mhm. This one’s gone.” [shakes bottle]

Clinician: [looks at bottle for 1 second, back to EHR] “Okay.”

#### Pattern 2: Dynamic Electronic Health Record Use Beyond the Current Medication List

To negotiate medication discrepancies, clinicians often navigated beyond the current medication list, using multiple sections within the note and the entire electronic chart, including past visit notes, notes from other settings, and test results. Clinicians multitasked, navigating and reading EHR sections while eliciting or listening to information from the patient. Clinicians also interspersed EHR multitasking with silent EHR use to concentrate on reviewing information or completing tasks. Patients often offered social talk breaking this silent EHR use and triggering clinician multitasking.

During visit P1 (female PCP, male patient, English-concordant; 40% EHR multitasking, 3% silent EHR use, 50% focused patient-clinician talk; 3.6 minutes), the clinician spent 3 minutes attempting to verify one medication, using multiple EHR sources of information (the medication list, three past clinician notes from the current and previous EHR), pill bottles, and patient history. Silent EHR use occurred when the clinician reviewed a previous visit note:

[Clinician sits facing EHR screen with hand on mouse. EHR screen is angled toward patient who sits facing clinician. Pill bottles are on the desk.]

Clinician: [looks at pill bottle] “Okay...You’re taking this one, the torsemide?” [shows patient pill bottle while clicking into the EHR current medication list]

Patient: [looks at pill bottle] “Yeah. Once a day.”

Clinician: [scrolling through medication list] “Is that a new one?”

Patient: “No.”

[Clinician looks at pill bottle, then back to medication list.]

Patient: [looking at clinician] “Why? It’s not on my list?”

Clinician: [begins typing in medication list search function] “It’s not, actually.”

Patient: [putting away backpack] “Then maybe I shouldn’t take it. It’s an old one.”

[Clinician looks down at pill bottle for one second, then back to medication list. Clinician exits out of medication list to History of Patient Illnesses section.]

Clinician: [clicking into list of previous clinician notes] “Hold on. When was the last time you saw these cardiac doctors?”

Patient: [looking at clinician while clinician scrolls down list of past visits] “Um, like two months ago. Yeah, two months ago.” [Clinician clicks into a cardiology visit note.] “I think I’ve got another appointment coming up.”

Clinician: [scrolls through note, pill bottle still in hand] “Okay, hold on, I’m going to try to read their note here.”

[5 seconds of silence as clinician looks at note. Patient looks down at hands.]

Patient: “How many years did it take for you to become a doctor like you are?”

[2 minutes of EHR multitasking; clinician clicks and scrolls through five different EHR sections while engaging in social talk with the patient.]

Clinician: [scrolls through note] “Okay here’s the deal. I can’t find...umm...” [highlights information in note] “...I cannot find this guy—“ [shakes pill bottle] “—in the notes from cardiology.” [turns to patient] “Okay, so we’re going to stop this, because this medicine acts the same as this one.” [picks up another pill bottle and shows patient] “So we don’t want two medicines doing the same thing. So you’re not going to use that anymore.”

#### Pattern 3: Focused Patient-Clinician Talk for Medication Counseling and Addressing Patient Concerns

Clinicians demonstrated different ways of interspersing focused patient-clinician talk with multitasking or silent EHR use. For example, clinicians used brief periods of focused talk to address patients’ medication or health concerns arising during medication reconciliation. In visit P2 (female PCP, male patient, Spanish-concordant; 51% EHR multitasking, 10% silent EHR use, 39% focused patient-clinician talk; 8.5 minutes), a clinician stopped multitasking on the EHR to address the patient’s worry about a side effect:

[Clinician sits with body toward the patient while clicking off medications in the EHR current medication list and examining pill bottles for 4 seconds.]

Patient: “For example, the one for the heart, they told me it could have a side effect that feels like arthritis.” [points to chest] “That’s what they told me.”

Clinician: [slightly turns head from screen, looks at patient] “Well, those medications for gout and arthritis aren’t really related to the heart. Don’t worry about that.”

[Patient hands clinician pill bottle. Clinician looks at it for <1 second and looks back to patient.]

Clinician: “And no—I don’t believe—It is true that gout could be a secondary effect of the medicine, but it’s not—” [turns head to EHR, pauses for 1 second] “Well, that’s not true.” [turns head back to patient] “The furosemide, the diuretic, could possibly—”

Patient: [hands clinician another pill bottle] “This one?”

Clinician: “Yeah, this one.”

Patient: “I’ve been taking this one for 25 years.”

Clinician: “But it’s not that the number of years affects the gout, it’s just that in the moment that you’re taking it you have greater risk of gout.”

Patient: [takes out other pill bottles] “Okay.”

Clinician: “Thank you for bringing these, I’m going to review these medications with the list in the computer.” [turns head to EHR screen]

The clinician then interspersed silent EHR use and EHR multitasking using pill bottles, updating the current medication list. While EHR multitasking, the clinician found out from the patient that he was no longer receiving colchicine for gout due to insurance limitations. After completing reconciliation of all pill bottles, the clinician readdressed the patient’s concern through focused talk:

[Clinician’s body oriented toward patient with full eye contact.]

Clinician: “So about the gout...It’s true that this—” [holds up pill bottle] “—has a side effect, and although your gout may be better controlled without this medicine, the rest of your body would be worse off without it—” [laughs] “—because you need this furosemide, and any medicine that removes water affects gout. So, I would like to continue the same with this medication—” [points to pill bottle] “—and I would like to increase—” [picks up other pill bottle] “—this medicine, the allopurinol, to prevent more gout attacks. What do you think of this plan? You agree, too?”

#### Pattern 4: Responding to Patient Concerns While Maintaining Electronic Health Record Use

Patients often revealed concerns about their medications and nonmedication topics during medication reconciliation, sometimes without clinician elicitation. Some clinicians responded by continuing to multitask, offering expressions of empathy or exploring patients’ concerns while continuing to use the EHR.

In visit P12 (female PCP, female patient, English-concordant; 76% EHR multitasking, 24% silent EHR; 1.6 minutes), the clinician addresses the patient’s concern verbally while continuing EHR tasks:

[Patient sits in a wheelchair adjacent to EHR monitor, facing the clinician. The clinician is walking toward EHR after sanitizing hands].

Patient: [concerned tone] “I was going to ask you, with my Ativan—” [clinician sits and faces EHR] “—if you can increase it.”

Clinician: [scrolls through History of Patient Illnesses] “Tell me about that. Tell me why you want to increase it.”

Patient: [looking at clinician] “Because...I’m under a lot of stress...”

Clinician: [scrolls through EHR list of visits, clicks into past visit note, inquisitive tone] “Mhm.”

Patient: “And what I was taking; it’s not working for me.”

Clinician: “Mm-hm.” [past visit note loads] “Tell me what else we’re doing to help.” [concerned tone] “I know you’re under a lot of stress.” [scrolls through note] “We’ve talked about, before, worrying about the side effects of the Ativan and I think there may be better medicines for you to take to deal with the stress.”

At times, clinicians did not respond to the patients’ concerns, continuing their multitasking or silent EHR use. In visit P5 (male PCP, female patient, English-concordant; 100% EHR multitasking; 5.6 minutes), the patient describes pain and depression which the clinician does not address during the visit:

[Clinician sits facing EHR screen, hand on mouse. Patient sits adjacent to monitor, facing clinician. Clinician has been EHR multitasking; discussing the patient’s medication-taking behaviors for 1 minute.]

Clinician: “...And, the other ones were the um, medication for your stomach.”

Patient: [looks away from clinician] “Yes, and also the other one is a cough syrup.” [concerned tone] “Yeah, sometimes I have—”

Clinician: [clicks medication in EHR current medication list, flat tone] “Okay.”

Patient: [looks back at clinician] “—tremendous pain.”

Clinician: [gaze on EHR, flat tone] “Okay, and are you still taking the Duloxetine? It’s like an antidepressant.” [glances <1 second at patient then back to EHR]

Patient: [gaze on clinician, concerned tone] “Yes. And when I take that in the morning, it makes me, you know.” [puts hand in a fist]

Clinician: [clicks in EHR current medication list, monotone] “Okay, you take that in the morning.”

Patient: [looking at clinician] “—I stop to cry.”

Clinician: [scrolls in medication list, flat tone] “Uh huh.”

Patient: [looking at clinician] “I stop to cry.”

Clinician: [shifts gaze to patient, flat tone] “You stop to cry?”

Patient: “Yes. When I take that for the uh—” [clinician nods, shifts gaze back to EHR] “—when I stop to take the medication for the depression, I get so sensitive. So sensitive.” [motions hand toward heart] “When I take my two pills in the morning, I am strong.” [laughs]

Clinician: [scrolling in medication list, flat tone] “Okay.”

Patient: “Yeah, I found out because I take notes also for my medication.”

[Clinician nods, maintaining gaze on EHR.]

#### Pattern 5: Clinicians Using Electronic Health Records to Engage Patients During Medication Reconciliation

In two encounters, clinicians with high levels of EHR use engaged patients, through screen sharing, transparent disclosure of EHR tasks, and shifting bodily orientation toward their patients.

In visit P10 (female PCP, male patient, English-concordant; 100% EHR multitasking; 2.2 minutes), the clinician shared her screen to review all nine of the patient’s medications, with the patient sitting next to her reviewing the EHR list actively:

Clinician: [clicks into current medication list] “So your meds...”

Patient: [looks at screen and reads] “Sildenafil, five tablets three times a day. Yes.”

Clinician: [clicks to check off medication] “You’re taking furosemide...”

Patient: [looks at clinician, then screen] “Which one is that?”

Clinician: “That’s the water pill.”

Patient: [looks at screen] “Uh, only if I need it. If I have swollen ankles.”

Clinician: [clicks to pull up text box] “How often are you taking it?”

Patient: [turns head from screen to clinician] “I haven’t taken it in probably 6 months.”

Clinician: [quickly turns toward the patient, makes eye contact, smiles, and turns back to screen] “Wow!” [turns back to the screen and types] “I’m going to leave it on your list but I’m going to put ‘as needed.’ How about that?”

Patient: [looks at screen] “Yeah. ‘As needed’ sounds good.”

In visit P6 (female PCP, male patient, English-concordant; 47% EHR multitasking, 4% silent EHR tasks, 13% non-EHR multitasking, 36% focused patient-clinician talk; 5.6 minutes), the clinician screen shares while multitasking and transparently explains the need to use EHR silently:

[EHR in triangle between clinician and patient. Clinician’s body is angled half toward patient and half toward EHR]

Clinician: [looking directly at patient] “I want to go over your medicines. Did you bring your box thing?”

Patient: “I did not bring it.”

Clinician: “OK” [glances at EHR med list, then back to patient] “Would you recognize the names?”

Patient: “Yeah! Yeah!”

Clinician: “OK let’s look through them.” [turns screen to share with patient] “Now I’m going to show you this thing. Can you see it?”

Clinician: [looking at screen with patient] “The furosemide—how are you taking that one?” [turns gaze to patient]

Patient: “I’m taking the one 80 pill in the morning and then at night.”

Clinician: “And how late do you take the second one?”

Patient: “The second one I take it around dinner time.”

Clinician: “OK good, because you don’t want to be up all night peeing...”

Patient: “Yeah...”

[They troubleshoot timing of the diuretic medication with focused talk]

### Conceptual Diagram: Multitasking Clinicians Balancing the Demands and Risks of Electronic Health Records and Communication Tasks During Medication Reconciliation

**Figure 2:**
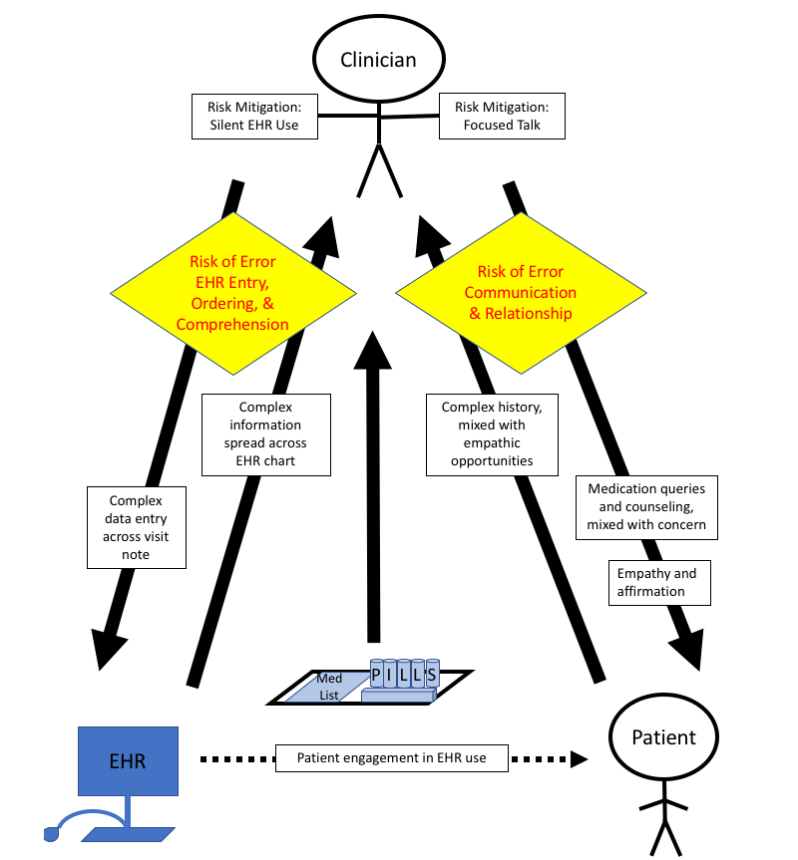
Conceptual diagram: multitasking clinicians balancing the demands and risks of electronic health records (EHRs) and communication tasks during medication reconciliation.

Based on this analysis, we developed a conceptual diagram representing the multitasking clinician balancing the cognitive and emotional demands posed by incoming information from multiple sources, attempting to synthesize and act on this information through EHR and communication tasks, and adopting strategies that may help mitigate the risks of this multitasking (Figure 2).

Because most clinicians multitask during medication reconciliation (pattern 1), this complex process is represented by solid black arrows demonstrating the input and output of information (1) between the clinician and the EHR, (2) between the clinician and the patient, and (3) between the clinician and the patient’s medication bottles or paper medication lists. To complete medication reconciliation, the clinician searches across the EHR chart to find, read, and process complex information entered by multiple members of clinical care teams (pattern 2). Meanwhile, clinicians navigate into multiple sections of the visit note to enter data relevant to medication reconciliation (pattern 2). At the same time, the clinician hears and processes complex patient histories about patients’ medication-taking behaviors and concerns, mixed with overt and subtle clues that offer empathic opportunities.

The clinician usually responds by eliciting more information and providing counseling, expressing concerns over medication discrepancies and suboptimal adherence (patterns 3 or 4). Sometimes, the clinician takes advantage of empathic opportunities to offer support and affirmation (patterns 3 and 5). Sometimes, the clinician does not recognize the empathic opportunities—potentially distracted by EHR tasks—or chooses to let the opportunities pass to continue completing medication reconciliation tasks (pattern 4).

The multitasking clinician has risks for error on two fronts, represented by the yellow diamonds. The clinician may make errors in EHR entry, ordering, and comprehension or errors in patient-clinician communication. Of note, communication errors may lead not only to risks to the patient-clinician relationship, but also errors in diagnosis and management if clues about patient symptoms or behaviors are not caught and addressed.

Consciously or unconsciously, clinicians may mitigate these errors by focusing on one interaction at a time. To mitigate the risk of EHR-related errors, clinicians may take periods of silence to focus solely on the EHR, investigating medication discrepancies with more complex and dynamic EHR use. To mitigate the risk of communication errors and relational damage, clinicians may cease EHR multitasking to focused patient-clinician talk.

This diagram’s focus is on the multitasking clinician. However, on rare occasions, patients actively engaged with the EHR use by watching their clinicians use the EHR or after receiving explicit invitation by clinicians to join the process. Potentially, this relationship-centered EHR use offers a third option for risk mitigation, allowing clinicians to feel more comfortable using the EHR with the patients at their side.

## Discussion

In this safety net study, patient-clinician visits exhibited interesting variations in the depth of medication reconciliation discussion and the patterns of EHR use to support that discussion. The absolute number and proportion of total medications addressed bore little relationship with the length of time, and rather than occurring in a “medication reconciliation block,” these discussions were interspersed with other content, including biomedical, psychosocial, and social talk. Overall, clinicians asked simple closed or open-ended questions about the patient’s medication-taking behaviors overall, with deeper investigation about only a subset of medications. Some clinicians employed the more detailed, patient-centered interviewing recommended for learning about a patient’s medication beliefs and burdens, but not about each medication on a given list. Of note, the longest medication reconciliation discussion lasted almost 10 minutes, addressing all four of the most relevant medications to that specialist, while leaving untouched the 10 other medications on the patient’s list. In a cross-sectional analysis, one cannot determine if the depth and length of these medication reconciliation discussions were affected by EHR use or the clinician’s pre-EHR approach to medication reconciliation, uninfluenced by the meaningful use requirement. Given that many relationships were more than 5 years, some clinicians may know some of this information from past visits. However, our results suggest that a comprehensive, patient-centered medication reconciliation interview with medically and psychosocially complex patients may be time-consuming for a safety net primary care or specialty clinician to conduct on their own.

Safety net patients experience barriers to patient-clinician communication and have higher medication reconciliation needs [5-10]. Limited health literacy is associated with poorer ability to interpret medication labels and their instructions, poorer ability to demonstrate taking medications, and inability to identify medications leading to a higher number of unreconciled medications [6-8]. Limited English proficiency, which often interacts with limited health literacy, is also associated with nonadherence to newly prescribed medications, errors in demonstrating how to measure doses of medications, and poorer knowledge of both chronic medications and medication changes on hospital discharge [31-35]. Our sample was also chronically ill with poor or fair quality of health and a high medication burden, also shown to be a risk factor for not taking medications on the current medication list [9,10]. Thus, medication reconciliation for safety net clinicians may be more important, but also more complex.

Meanwhile, multitasking EHR use comprised almost half of medication reconciliation time, a higher proportion of time than in the visits as a whole [17]. Many clinicians may not believe they are multitasking when updating the EHR medication list while talking to patients because both tasks are concordant with the goals of reconciling medications [36]. However, research in cognitive psychology suggests that the act of reading or entering computer data while listening or talking with another person may increase both the risk of errors and the time required to complete each task [16]. Although this risk may be lower when a patient is affirming the accuracy of the current medication list, this risk increases when the clinician is conducting more complex cognitive steps required to negotiate medication discrepancies.

Clinical multitasking predated EHRs, with clinicians reviewing paper charts or patient pill bottles while interviewing patients. EHRs have the potential to reduce errors overall [37-39] by reducing the cognitive difficulties in this work by synthesizing and organizing information in accessible, usable formats, supporting clinical decision making, and offering new information that was previously unavailable, such as medication refill data. However, research is increasingly recognizing the risk of technology-induced errors arising from a technology’s design and development, implementation and customization, and resultant human-computer interactions and sociotechnical work processes [19,20].

Our study suggests that clinician multitasking is associated with important risks of errors in communication, which pose not only dangers to the relationship but also the accurate diagnosis and management of the patient’s medical and psychosocial needs. As seen in pattern 4, sometimes patients express medication and nonmedication concerns that would require deeper exploration. These concerns may signal suboptimal adherence, an undiagnosed or undertreated medical or psychosocial condition, and a need for empathy and reassurance from the clinician.

As the conceptual diagram depicts, multitasking clinicians using an EHR for medication reconciliation must cope with the cognitive and emotional burdens of this work while managing many other tasks. When patients disclose nonadherence or offer their concerns about medications or other topics, through overt or more subtle clues, clinicians have a series of cognitively challenging tasks. First, they must recognize the cues, which may be more challenging during EHR multitasking when their gaze and attention is not focused on the patient. Second, if they recognize the clues, clinicians must choose whether to respond through further exploration at that time (with focused talk or continued multitasking) or by deferring the exploration until after completing their medication reconciliation tasks. If clinicians respond at the time, they risk making a mistake, both in the EHR tasks and in their communication. If they do not respond at the time, they risk missing important patient engagement opportunities, to the detriment of both patient satisfaction and patient care. This study offered examples of all those patterns and the transitions across them, but this study cannot reveal how many of the clinicians’ choices were intentional to mitigate risk or subconscious. We also do not know what these patients would have preferred and what they felt about their clinicians’ communication and EHR use behaviors in those moments.

Finally, a few clinicians in this sample exemplified behaviors of harnessing EHRs to further relationship-centered communication [40]. In addition to sharing the EHR screen through inclusive positioning [41], these clinicians used body language, eye contact, affective tone of voice, and empathy statements to elicit and respond to patients’ concerns. In those cases, patient-centered medication reconciliation addressed the majority of these patients’ medications in less than 6 minutes. This kind of EHR use likely has many facilitators, including the clinicians’ communication style, their existing relationships with their patients, their computer and EHR proficiency, and the encounter room positioning. However, these examples lend support for recent efforts to teach real-time EHR use during patient-clinician encounters [42], since shifting all EHR documentation to nonvisit times or other team members may not be possible or sustainable for most clinicians. Clinicians need additional training and support on how to transition intentionally between multitasking, silent EHR use, and focused clinician-patient talk when appropriate to the situation, using strategies to communicate these transitions transparently to their patients.

This analysis also adds to the growing literature about newer generation EHRs in the United States under the meaningful use incentives programs, particularly in a safety net primary and specialty care setting. This study adds to the call for clinicians, health systems, and policymakers to redefine medication reconciliation to acknowledge the multiple levels of medication reconciliation that incorporates the patient’s full perspective, including eliciting opportunities to describe and reduce the physical, emotional, and economic burdens of medication regimens [3]. Better measures of high quality medication reconciliation, incorporating the patient’s and clinician’s perspectives, are needed. Mandates and incentives to promote medication reconciliation are insufficient for promoting high quality medication reconciliation and may increase the risk of technology-induced errors associated with currently designed and implemented EHRs. Health information technology should be designed and developed, using human factors and systems engineering framework, to facilitate engagement across the appropriate members of the health system care team, community pharmacists, family members and caregivers, and the patient.

This study’s limitations should be considered. First, our results may be affected by volunteer bias among clinicians or patients. Second, the sampled visits occurred early after EHR implementation and may not represent medication reconciliation after clinicians spent more time using the EHR, although other researchers have found that early EHR and communication behaviors may be similar to those measured later after implementation [12]. Third, this cross-sectional study was not designed to study process or clinical outcomes, including clinical slips or mistakes, and cannot be used to make causal inferences. Fourth, our study was not intended to identify the number and severity of discrepancies uncovered during medication reconciliation, which would be an important area for discovery in future research about EHR multitasking. Finally, as a qualitative study in a single safety net network, we are not able to develop theories based on particular provider or patient subgroup characteristics, and our findings may not be generalizable to other settings. The study strengths are its inclusion of primary care and specialty care providers, physicians and nurse practitioners, and a medically, socioeconomically, and linguistically diverse safety net population.

In summary, the multitasking clinician balances the cognitive and emotional demands posed by incoming information from multiple sources, attempts to synthesize and act on this information through EHR and communication tasks, and adopts strategies of silent EHR use and focused patient-clinician talk that may help mitigate the risks of multitasking. Future studies should explore diverse patient perspectives about clinician EHR multitasking, clinical outcomes related to EHR multitasking, and human factors and systems engineering interventions to improve the safety of EHR use during the complex process of medication reconciliation.

## Appendix

Multimedia Appendix 1 Codes associated with conceptual diagram: multitasking clinicians balancing the demands and risks of EHR and communication tasks during medication reconciliation.

## Abbreviations

CMS: Centers for Medicare and Medicaid Services
EHR: electronic health record
IQR: interquartile range
PCP: primary care physician
