# Supplementary figures and images for: Finding Meaning in Medication Reconciliation Using Electronic Health Records: Qualitative Analysis in Safety Net Primary and Specialty Care
Source: JMIR Med Inform. 2018 May 7;6(2):e10167. doi: 10.2196/10167 (PMC5962827; doi:10.2196/10167)

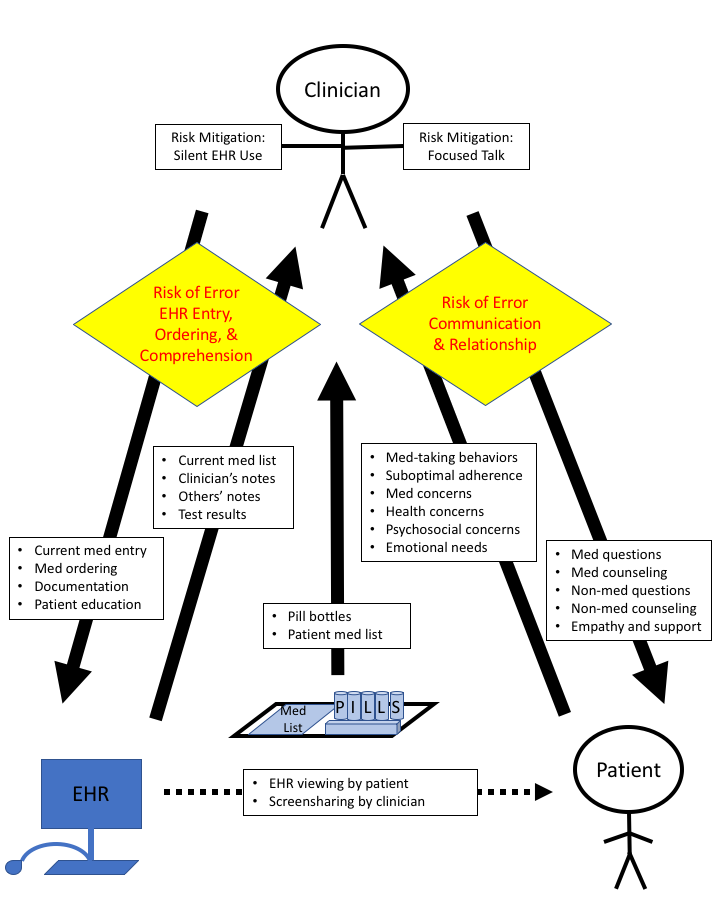

Supplement: Multimedia Appendix 1 [file medinform_v6i2e10167_app1.png]
